# Negligible effect of surgeon experience on the accuracy and time to perform unrestricted caliper verified kinematically aligned TKA with manual instruments

**DOI:** 10.1007/s00167-022-06939-y

**Published:** 2022-04-02

**Authors:** Stephen M. Howell, Alexander J. Nedopil, Maury L. Hull

**Affiliations:** 1 Department of Biomedical Engineering, University of California at Davis, 451 E. Health Sciences Drive, Room 2303,, Davis, CA 95616 USA; 2 Department of Orthopaedic Surgery, König-Ludwig-Haus, University of Würzburg, Würzburg, Germany

**Keywords:** Knee arthroplasty, Kinematic alignment, Accuracy, Manual instruments

## Abstract

**Purpose:**

Surgeons performing total knee arthroplasty (TKA) are interested in the accuracy and time it takes to make the four femoral resections that determine the setting of the femoral component. A method for quantifying the error of each resection is the thickness, measured by a caliper, minus the femoral target. The present study tested the hypothesis that the mean deviation of the resection from the femoral target, the percentage of resections with a deviation of ± 0.5, 1.0, 1.5, and 2.0 mm, and the time to complete the femoral cuts were not different between experienced (E) and less-experienced (LE) surgeons performing unrestricted caliper verified kinematically aligned (KA) TKA with manual instruments.

**Methods:**

This study analyzed intraoperative verification worksheets from 203 patients treated by ten E surgeons and 58 patients treated by four LE surgeons. The worksheet recorded (1) the thickness of the femoral target for the distal medial (DM), distal lateral (DL), posterior medial (PM), and posterior lateral (PL) resections and the caliper thickness of the resections with a resolution of 0.5 mm, and (2) the time to complete them. The most accurate resection has a mean difference ± standard deviation of 0 ± 0.0 mm.

**Results:**

The accuracy of the 1044 initial resections (261 patients) was significantly closer to the femoral target for E vs. the LE surgeons: 0.0 ± 0.4 vs. − 0.3 ± 0.5 for the DM, 0.0 ± 0.5 vs. − 0.4 ± 0.6 for the DL, − 0.1 ± 0.5 vs. − 0.2 ± 0.5 PM, and − 0.1 ± 0.5 vs. − 0.4 ± 0.6 for the PL resections (*p* ≤ 0.0248). E surgeons completed the femoral resections in 12 min; 5 min faster than LE surgeons (*p* < 0.0001).

**Conclusions:**

Because the mean difference in femoral resections with manual instruments for E vs. LE surgeons was < 0.5 mm which is within the caliper’s resolution, differences in accuracy were not clinically relevant. Surgeons exploring other alignment options and robotic, navigation, and patient-specific instrumentation might find these values helpful when deciding to change.

**Level of evidence:**

III; case–control study.

## Introduction

Surgeons performing total knee arthroplasty (TKA) and considering different alignment options and instrumentation are interested in the accuracy and time it takes to make the distal and posterior femoral bone resections that determine the setting of the femoral component [7]. Technology proponents argue that robotic, navigation, and patient-specific instrumentation more accurately hit the femoral target than manual instruments [12, 14, 22, 28]. Whereas manual instrument proponents argue that technology lengthens the operation, adds expense, and induces stacked errors arising from transforming images into a 3D model, planning the resection planes, and registering instruments [1, 4, 5].

Unrestricted caliper verified kinematic alignment (KA) sets the femoral component coincident to the patient’s pre-arthritic joint lines, a sine qua non for restoring the native limb alignment and tibial compartment forces without releasing ligaments [17, 18, 23, 24]. The essential instrument is an inexpensive caliper, which measures the thickness of the distal and posterior resections enabling correction when they deviate from the femoral target before implantation [4, 5].

Surgeons depend on accuracy values to compare new instrumentation. One statistic is the deviation of the resection thickness from the femoral target, and 0 ± 0.0 mm (mean ± standard deviation) indicates the highest accuracy. Another is the percentage of resections within a deviation of ± 0.5, 1.0, 1.5, and 2 mm.

There are no studies of the effect of surgeon experience on the accuracy of performing unrestricted caliper verified KA using manual instruments, which is of interest to surgeons exploring alignment options and robotic instrumentation. Accordingly, the present study evaluated the femoral resections from 208 patients performed by ten experienced surgeons (i.e., greater than 50 KA TKAs in their career) and 58 patients treated by four less-experienced surgeons. The hypothesis was that the mean deviation of the resections from the femoral target, the percentage of resections with a deviation of ± 0.5, 1.0, 1.5, and 2.0 mm, and the time to complete the femoral cuts were not different between experienced and less-experienced surgeons.

## Materials and methods

An institutional review board approved this retrospective study (IRB 00054838) of deidentified prospectively collected data obtained and processed in the following manner. Information on each subject sent by the treating surgeons to the investigators was recorded in such a manner that the identity of the human subjects could not be ascertained either directly or through identifiers linked to the subjects. Furthermore, the investigators did not contact subjects, and the investigators did not reidentify subjects. Because the IRB deemed this secondary research of de-identified information, consent was not required.

**Fig. 1:**
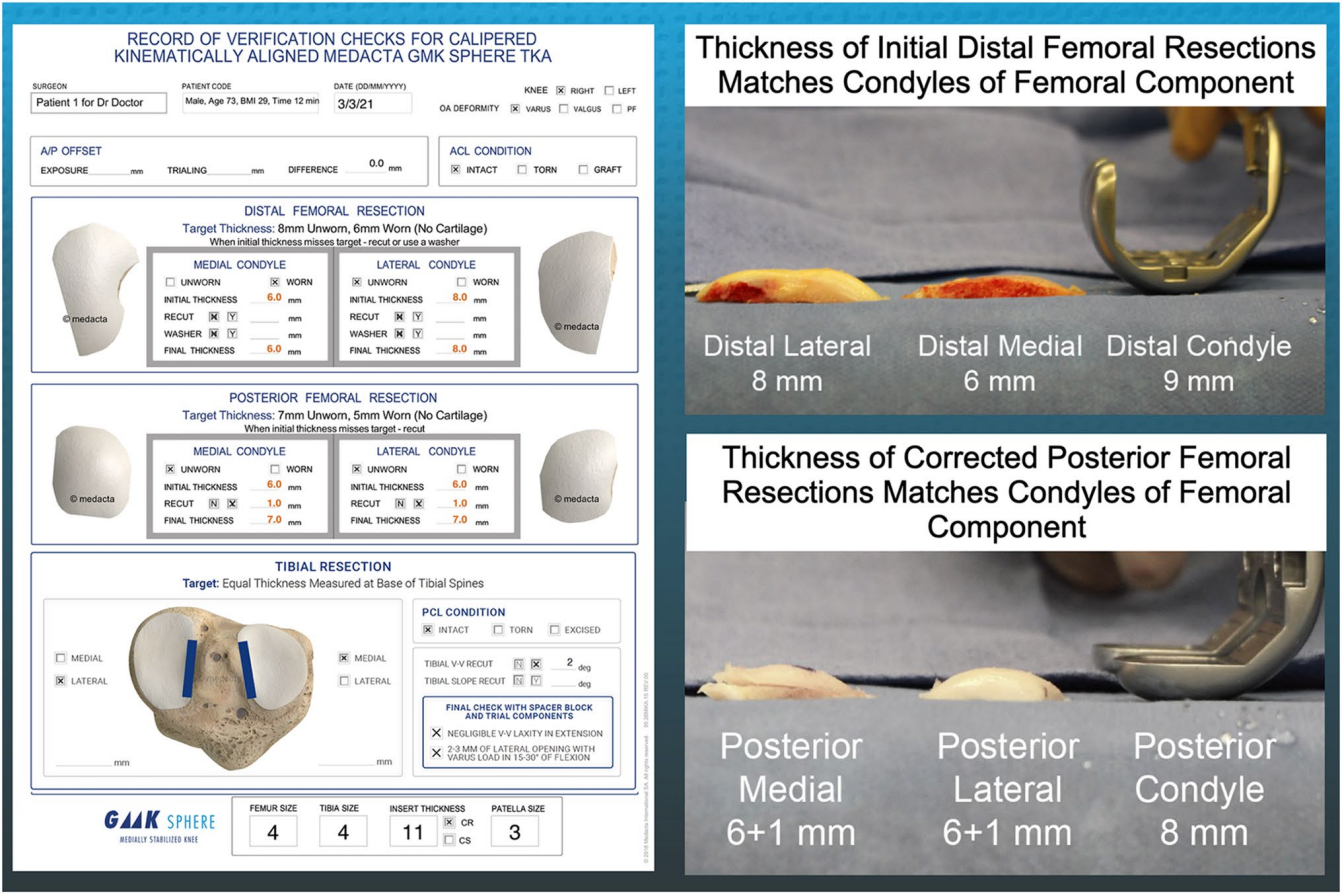
Composite of images shows the intraoperative verification worksheet of a patient including entries for patient number, surgeon name, sex, age, BMI, time to complete corrected femoral cuts, date of surgery, right or left knee, type of primary deformity (varus, valgus, or patellofemoral), condition of ACL, plan thickness of distal medial and lateral and posterior medial and lateral femoral resections, initial and corrected caliper thickness of each femoral resection (left) and the recordings of the thicknesses of the distal and posterior femoral bone resections compared to the thicknesses of the condyles of the femoral component (right)

Between March 2021 and June 2021, fourteen surgeons provided verification worksheets from consecutive patients treated for primary knee osteoarthritis who did not have avascular necrosis, septic arthritis, or a prior intra-articular fracture (Fig. 1). In addition, there were no exclusions based on the knee's preoperative deformity and flexion contracture severity. Each patient underwent an unrestricted caliper verified KA TKA using manual instruments and a previously described technique (Medacta International, (Medacta International, www.medacta.com) [2]. Excluded were patients with prior intra-articular fracture, bone loss from avascular necrosis, and septic arthritis. Data recorded on the verification worksheet included a deidentified patient number, surgeon name, age, BMI, sex, date of surgery, right or left knee, sex, type of primary deformity (varus, valgus, or patellofemoral), condition of ACL, plan thickness of the distal and posterior medial and lateral femoral resections, initial and corrected caliper thickness of each femoral resection, time to complete these femoral resections, insert constraint (i.e., posterior cruciate ligament retaining or substituting), and component sizes (Table 1). Each surgeon recorded the time in minutes from the incision to completion of the femoral cuts and subtracted out the time for performing the patella resection before femoral preparation.

**Table 1:** Comparision of a series of patient characteristics and knee conditions and the results of either a Student’s *T* test or a Fisher’s Exact Test to compute the significance of differences between the 203 patients treated by ten experienced and 58 patients treated by four less-experienced surgeons

Patient characteristics				Significance
Age (years)	Mean	67.5	66.3	NS, *p* = 0.1967*
	Std Dev	9.6	9.1	
Body mass index (kg/m^2^)	Mean	31.1	30.8	NS, *p* = 0.3636*
	Std Dev	5.8	5.0	
Right vs. left				NS, *p* = 0.3733^#^
Left		102	25	
Right		101	33	
Sex				NS, *p* = 0.3771^#^
Female		112	36	
Male		91	22	
OA deformity				NS, *p* = 0.2804^#^
Varus		153	43	
Valgus		37	14	
Patellofemoral		13	1	
ACL condition				NS, *p* = 0.7562^#^
Intact		126	37	
Torn		47	16	
ACL reconstructed		7	1	
Type of insert constraint				NS, *p* = 1.0000^#^
Posterior cruciate ligament retaining (CR)		131	37	
Posterior cruciate ligament substituting (CS)		72	21	

**Fig. 2:**
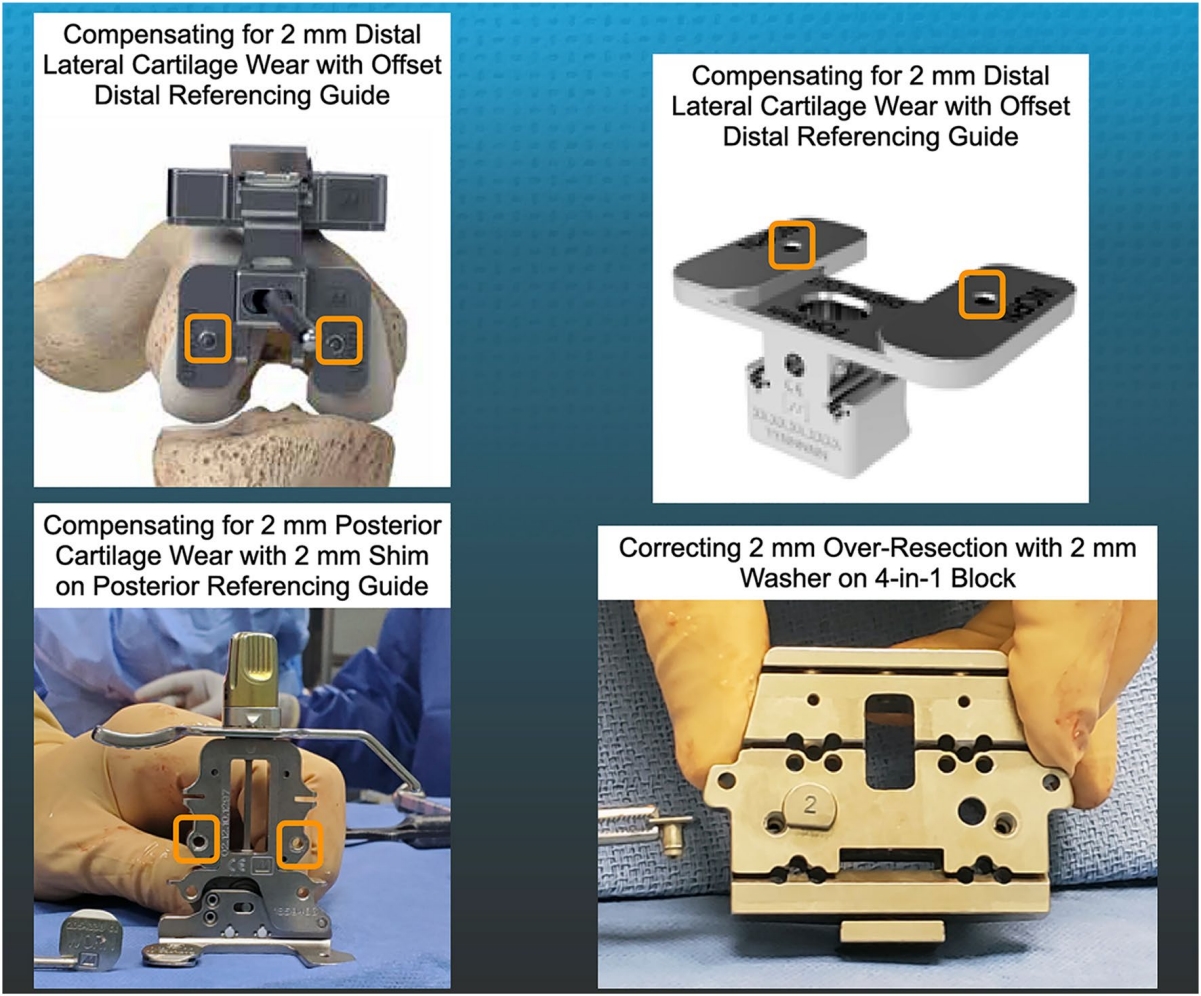
Composite of images shows the manual instruments used to make the distal and posterior femoral resections. The features include an offset distal referencing guide with two holes (orange squares) for compression screws (upper left and right), a posterior referencing guide set at 0° with small, medium, and large width posterior feet with two holes (orange squares) for compression screws and removable shims to compensate for 2 mm of distal and posterior cartilage wear (lower left), and a washer, available in 1 and 2 mm (shown) thicknesses, to correct for an over-resection of a distal femoral condyle (lower right)

The following steps describe the use of manual instruments to set the femoral component’s varus–valgus orientation and proximal–distal position coincident to the patient’s pre-arthritic distal femoral joint line (Fig. 2) [2]. The basis for setting the distal and posterior femoral resection is knowing that the varus and valgus Grade II to IV Kellgren–Lawrence osteoarthritic femoral condyles have negligible bone wear at 0° and 90° flexion and that the mean full-thickness cartilage wear approximates 2 mm [8]. With the exposed knee in 90° of flexion, the pattern of cartilage wear is examined on the articular surface of the femur without referring to a knee radiograph. Partially worn cartilage is removed to subchondral bone with a ring curette. A positioning rod is inserted 10 cm into the femoral metaphysis through a drill hole made perpendicular to the distal femoral joint line. Distal femoral resections were performed to kinematically align the distal joint line of the femoral component coincident with the pre-arthritic distal joint line [11]. The thickness of each resection should equal the femoral target of the thickness of the condyle of the femoral component minus 1 mm for the thickness of the kerf of the saw cut and minus 2 mm for worn cartilage. The thicknesses of the distal femoral resections were measured with a caliper with a resolution of 0.5 mm and recorded on the verification worksheet. The surgeon recorded when the femoral target was missed by more than ± 0.5 mm. The surgeon then recorded whether they corrected the deviation from the femoral target and the amount of correction (Fig. 1). When a resection was less than the femoral target or under-resected, the initial resection was corrected by removing additional bone by either 1) redirecting the saw blade through the cutting block, 2) using a 1–2 mm recut guide, or 3) free-handing the cut until the resection is within ± 0.5 mm of the femoral target.

The following steps describe the setting of the femoral component’s internal–external orientation and anterior–posterior coincident to the patient’s pre-arthritic posterior femoral joint line (Fig. 2). With the tip of a knife the thickness of the cartilage of the posterior condyles is checked. When the cartilage is intact the posterior referencing guide set at 0° of rotation is compressed against the distal femur with a threaded pin. When the cartilage is worn, a 2 mm shim was inserted between the foot of the referencing guide and the posterior condyle. Be aware that the lateral femoral condyle is not hypoplastic in those knees with valgus osteoarthritis, so that restoration of the pre-arthritic joint line does not require an adjustment on the lateral femoral condyle [3]. The correct size 4-in-1 cutting block was selected. When a distal resection was 1 or 2 mm greater than the femoral target or over-resected, a 1- or 2-mm washer was placed on the 4-in-1 cutting block. The washer displaces the 4-in-1 cutting block distally, which creates a shallow anterior and posterior chamfer cuts that maintains a gap between the over-resected distal femoral resection and the femoral component that is filled with cement. The posterior femoral resections were made, and each thickness was measured with the calipers and recorded on the verification worksheet. The surgeon recorded when the femoral target was missed by more than ± 0.5 mm, and the amount of correction. The anterior, anterior chamfer, and posterior chamfer resections were made after the distal and posterior femoral resections.

### Statistical analysis

As an example, a power analysis was computed using a 1.0 mm mean difference in the target thickness minus the caliper measurement of the distal medial femoral resection between the patients assigned to the experienced and less experienced surgeon groups. A conservative 1 mm mean difference was selected, because a 2 mm increase in the level of the joint lines stiffens the knee by doubling the medial and lateral tibial compartment forces from native [23]. Assuming a Type I error (alpha) of 0.05, a power (1-beta) of 0.90, and a standard deviation of ± 1.0° the power analysis computed a sample size of 22 patients per experienced and less experienced surgeon groups.

Data on the verification worksheet were compiled, assigned to either an experienced surgeon group or a less-experienced surgeon group, and analyzed using statistical software (JMP^®^ Pro 16.0.0, www.jmp.com, SAS, Cary, NC, USA). The error for each femoral resection was the thickness, measured by a caliper, minus the femoral target (− under / + over resection), and the mean difference ± standard deviation described the accuracy. For the distal medial, posterior medial, distal lateral, and posterior lateral resection, a Student’s *T* test determined whether the mean difference from the femoral target was different between the experienced and less experienced surgeons. For each resection and the corrected one, the percentage of the total number of resections that fell within categories of within ± 0.5, ± 1.0, ± 1.5, and 2.0 mm of the femoral target were computed, and a Fisher’s Exact Test determined whether the proportions within each category were different between experienced and less experienced surgeons. A Student’s *T* test determined whether the mean time to complete the femoral resections was significantly different between the experienced and less experienced surgeons. Significance was *p* < 0.05.

## Results

The analysis of the patient and knee characteristics showed no significant differences between the 203 patients treated by the ten experienced surgeons and the 58 patients treated by the four less-experienced surgeons (Table 1). Experienced surgeons more accurately performed the initial distal and posterior femoral resections with mean differences closer to the femoral target than less-experienced surgeons (*p* = 0.0281 to < 0.0001) (Table 2). Except for the posterior medial femoral resection, the experienced surgeons had a higher percentage of resections and corrected ones within ± 0.5 and ± 1.0 mm of the femoral target than the less-experienced surgeons (Table 3). Experienced surgeons completed the femoral resections in a mean of 12 ± 3 min, which was 5 min faster than the time of 17 ± 5 min for the less experienced surgeons (*p* < 0.0001).

**Table 2:** For the experienced and less-experienced surgeons, the mean and standard deviation in millimeters (mm) of the target minus the initial femoral resection and those resections with a significant difference between surgeons’ level of experience as determined by the Student’s *T* test are shown

	Surgeon’s level of experience		Significance
	Experienced (> 50 Calipered KA TKA)	Less experienced (< 50 Calipered KA TKA)	
Distal medial target minus initial femoral resection in mm			
Mean	− 0.0	− 0.3	*p* < 0.0001*
Std Dev	0.4	0.5	
Distal lateral target minus initial femoral resection in mm			
Mean	− 0.0	− 0.4	*p* < 0.0001*
Std Dev	0.5	0.6	
Posterior medial target minus initial femoral resection in mm			
Mean	− 0.1	− 0.2	*p* = 0.0281*
Std Dev	0.5	0.5	
Posterior lateral target minus initial femoral resection in mm			
Mean	− 0.1	− 0.4	*p* = 0.0002*
Std Dev	0.5	0.6

**Table 3:** For the experienced and less-experienced surgeons, the proportions of initial and corrected femoral resections within different ranges from the KA target, and the results of a Fisher’s Exact Test that computed the significance of differences between the 203 patients treated by 10 experienced and 58 patients treated by four less-experienced surgeons are shown

	Surgeon’s level of experience		
	Experienced (> 50 Calipered KA TKA)	Less experienced (< 50 Calipered KA TKA)	
Initial distal medial femoral resection hit target?			NS, *p* = 0.1028^#^
Yes (within ± 0.5 mm of target)	89%	81%	
No (within ± 1 mm of target)	99%	98%	
No (within ± 2 mm of target)	100%	98%	
No (within ± 3 mm of target)	0%	2%	
Corrected distal medial femoral resection hit target?			NS, *p* = 0.1129^#^
Yes (within ± 0.5 mm of target)	95%	100%	
No (within ± 1 mm of target)	100%	100%	
Initial distal lateral femoral resection hit target?			*p* < 0.0332^#^
Yes (within ± 0.5 mm of target)	89%	76%	
No (within ± 1 mm of target)	97%	93%	
No (within ± 1.5 mm of target)	98%	97%	
No (within ± 2 mm of target)	100%	100%	
Corrected distal lateral femoral resection hit target?			*p* < 0.0105^#^
Yes (within ± 0.5 mm of target)	95%	83%	
No (within ± 1 mm of target)	100%	100%	
Initial posterior medial femoral resection hit target?			NS, *p* = 0.0648^#^
Yes (within ± 0.5 mm of target)	89%	79%	
No (within ± 1 mm of target)	97%	98%	
No (within ± 2 mm of target)	100%	100%	
Corrected posterior medial femoral resection hit the target?			NS, *p* = 0.7600^#^
Yes (within ± 0.5 mm of target)	94%	93%	
No (within ± 1 mm of target)	100%	100%	
Initial posterior lateral femoral resection hit target?			*p* < 0.0091^#^
Yes (within ± 0.5 mm of target)	91%	79%	
No (within ± 1 mm of target)	98%	91%	
No (within ± 1.5 mm of target)	99%	0%	
No (within ± 2 mm of target)	100%	100%	
Corrected posterior lateral femoral resection hit the target?			N/A
Yes (within ± 0.5 mm of target)	93%	95%	
No (within ± 1 mm of target)	99%	100%	
No (within ± 1.5 mm of target)	100%	100%	

#Fisher’s Exact

## Discussion

The most important findings of the present study were that the difference between the accuracy of experienced and less experienced surgeons performing KA with manual instruments was smaller than the 0.5 mm resolution of the caliper and clinically unimportant, and experienced surgeons completed the resections 5 min faster. In addition, the accuracy of the experienced and less experienced surgeons cutting the resection to the femoral target was comparable or better than reported values from studies of mechanical alignment using robotic and patient-specific instrumentation [4–7, 13, 22, 26, 30] (Table 4). Hence, this lends confidence to those surgeons considering a transition from MA and robotic instrumentation to unrestricted caliper verified KA that their lack of experience will not seriously compromise accuracy when performing the femoral resections with manual instruments.

**Table 4:** Comparison of the accuracy of experienced and less experienced surgeons performing each femoral resection using caliper verified KA with manual instruments to two robotic brands and patient-specific instrumentation (PSI)

Study	Alignment	Surgeons (knees)	Instrumentation	Accuracy of the distal and posterior resections is the mean (± SD) deviation from the femoral target			
				Distal medial	Distal lateral	Posterior medial	Posterior lateral
Present Study	KA (experienced)	10 (203)	Manual	0.0 (± 0.4)	0.0 (± 0.5)	− 0.1 (± 0.5)	− 0.1 (± 0.5)
Present Study	KA (< experienced)	4 (58)	Manual	0.3 (± 0.5)	0.4 (± 0.6)	− 0.2 (± 0.5)	− 0.4 (± 0.6)
Li et al. ([6])	MA	1 (36)	Robot MAKO	0.4 (± 0.6)*	0.5 (± 0.7)*	0.6 (± 0.8)*^#^	0.7 (± 0.8)*^#^
Seidenstein et al. ([22])	MA	4 (15 cadaver)	Robot ROSA	0.7 (± 0.7)*^#^	0.7 (± 0.7)*	0.6 (± 0.5)*^#^	0.6 (± 0.5)*^#^
Wernecke ([29])	MA	2 (118)	PSI	0.9 (± 1.3)*^#^	0.9 (± 1.3)*^#^	1.5 (± 2.1)*^#^	0.8 (± 1.2)*^#^

A Student's *T* test determined which of the KA distal and posterior resections performed with manual instrumentation by the ten experienced (* indicates *p* < 0.0001) and four less experienced surgeons (^#^indicates *p* = 0.0296 to < 0.0001) were significantly more accurate than robotic and PSI

One explanation for the high accuracy of performing the initial femoral resections is that the KA reference landmarks of the distal and posterior femoral joint lines are easily accessible and reliably registered with manual instruments, assuming adequate knee exposure. Furthermore, compressing the distal and posterior femoral guides directly to the femur with screws simultaneously plans and executes the distal and posterior femoral resections eliminating errors from converting images into a 3D model and computer planning of resection planes [4, 7].

Results of robotic studies summarized in a meta-analysis describe high accuracy in achieving MA to the femoral head and ankle [31]. However, unrestricted KA does not need the robotic accuracy of referencing the distant hip and ankle centers. Instead of technology, KA surgeons rely on the caliper to detect a resection deviation from the femoral target. Correcting a distal over-resection is achieved by modifying chamfer cuts and filling any gaps with cement [4, 5]. In contrast, correcting a posterior over-resection is more challenging, which some surgeons left under-corrected. Even with this limitation, 91–100% of the corrected distal and posterior femoral resections were within ± 1.0 mm of the femoral target, which is comparable if not a higher percentage to target than reported values of robotic, navigation, and patient-specific instrumentation [4–7, 13, 22, 26, 30] (Table 4).

It took 12 and 17 min for experienced and less-experienced surgeons to perform the initial femoral resections and correct any deviations from the femoral target using manual instruments, which is more efficient than robotic and navigation instrumentation. In addition, manual instruments require less setup time than robotic and navigation systems that insert large diameter pins to rigidly attach marker arrays for registration with the risk of periprosthetic fracture [1, 25, 27]. In contrast, caliper verified KA with manual instrumentation sets the distal and femoral resections after assessing areas of cartilage wear on the femur without the potential error, expense, and inconvenience from referring to a pre-operative radiograph or advanced imaging study [8]. In addition, 3-D and 2-D image analyses showed close coalignment of the femoral component set with caliper verified KA and the cylindrical or transverse flexion–extension axis by accurately restoring the patient’s pre-arthritic distal and posterior femoral joint lines [9, 10, 17].

The present study has several limitations. First, the accuracy of the resections reported in the present study apply to only one system of manual instruments explicitly designed for caliper verified KA that used compression screws to securely fix the distal and posterior referencing guides to the femur. In addition, the surgeons recognized over and under-resection deviations from the femoral target and corrected them before implanting the femoral component. Thus, surgeons adapting MA manual instruments for KA should consider measuring the resection deviation with a caliper and computing the accuracy for their technique. A second limitation is that a literature search found only two robotic systems that reported the deviation between the distal and posterior femoral resections and the femoral target. Other robotic systems might have different accuracy values, and manufacturers should analyze caliper measurements of the resections and report them. Finally, the technique of caliper verified KA with manual instruments cannot quantitatively measure knee laxity, which proponents of robotic and navigation instrumentation consider valuable. Relying on laxity measurements to balance a TKA is confounded as native laxities vary widely and are patient-specific, so striving for a mean or gold standard value is not physiologic [19, 21]. In addition, restoring the native laxities can be associated with an elevation in tibial compartment forces high enough to cause knee stiffness, which occurs with small, 1–2-degree deviations in the setting of the femoral and tibial components away from the patient’s pre-arthritic joint lines [15, 16, 20]. Fortunately, unrestricted caliper verified KA, retaining the posterior cruciate ligament, and not releasing other ligaments restores native laxities and medial and lateral tibial compartment forces without using an intraoperative tibial force sensor.

## Conclusions

Surgeons interested in performing unrestricted caliper verified KA using manual instruments might find the present study’s accuracy values for performing the initial femoral resections helpful when deciding to change.
